# Supplementary material for: Effects and Mechanisms of Exercise on Brain-Derived Neurotrophic Factor (BDNF) Levels and Clinical Outcomes in People with Parkinson’s Disease: A Systematic Review and Meta-Analysis
Source: Brain Sci. 2024 Feb 21;14(3):194. doi: 10.3390/brainsci14030194 (PMC10968162; doi:10.3390/brainsci14030194)
Supplement: Supplementary file 1 [file brainsci-14-00194-s001.zip › brainsci-2834366-supplementary.pdf]

Supplementary S1. Meta-analytic results of studies of exercise-induced BDNF in human PD [40,42-46, 52-55, 61].

Supplementary S1		Meta-analytic results of studies of exercise-induced BDNF in human PD (year of publication)								
		Frazzitta (2014)	Zoladz (2014)*	Angelucci (2016)*	Sajatovich (2017)**	O' Callaghan (2020)	Szymura (2020)	Freidle (2022)	DiCagno (2023)	Meta-analytic result
Meta-analysis	Kaagman	Yes	No	No	No	Yes	Yes	Yes	Yes	N=216<br>Statistically significant<br>SMD 1.20, CI=0.53 to 1.87
	Rotondo (2023)	Yes	No	No	No	No	Yes	Yes	No	N=180<br>Statistically significant<br>MD 5.99, CI=0.15 to 11.83
	Li (2023)	Yes	No	No	No	Yes	Yes	Yes	No	N=192<br>Statistically significant<br>Hedges g=0.54, CI=0.10 to 0.98
	Johansson (2022)	Yes	Yes	Yes	No	No	No	No	No	N=36<br>Statistically not significant
	Hirsch (2018)	Yes	No	No	Yes	No	No	No	No	N=52<br>Statistically significant<br>SMD 2.06, CI=1.36 to 2.76

Table note: *=uncontrolled pre-post test design; **=comparative effectiveness design; N=number of subjects entered into meta-analysis calculation; SMD=standardized mean difference; MD=mean difference; CI=95 percent confidence interval; %=percent; Hedges g is a measure of effect size. Yes=study included in meta-analysis; No=study not included in meta-analysis
